# What to expect from primary inferior oblique overaction after esotropia surgery: Primary inferior oblique overaction in esotropia

**DOI:** 10.1186/s12886-023-03256-x

**Published:** 2023-12-15

**Authors:** Paulo Freitas-da-Costa, Hélio Alves, Renato Santos-Silva, Fernando Falcão-Reis, Jorge Breda, Augusto Magalhães

**Affiliations:** 1 Department of Ophthalmology, São João University Hospital Center, Porto, Portugal; 2 Department of Surgery and Physiology, Faculty of Medicine, University of Porto, Porto, Portugal; 3 Anatomy Unit, Department of Biomedicine, Faculty of Medicine, University of Porto, Alameda Prof. Hernâni Monteiro, Porto, 4200-319 Portugal

**Keywords:** Esotropia, Strabismus, Ocular motility disorder, Inferior oblique muscle, Eye movements

## Abstract

**Background:**

Overelevation in adduction is common in patients with primary esotropia. This study evaluates the variation in ocular motility pattern in patients with primary inferior oblique (IO) muscle overaction after esotropia surgery.

**Methods:**

The medical records of consecutive patients who underwent surgery for infantile, partially accommodative, and basic esotropia over eleven years and had at least one year of follow-up were reviewed. Patients with primary inferior oblique muscle overaction (IOOA) presented at baseline or during follow-up were selected and divided according to the first surgery performed concurrently with horizontal rectus surgery: without IO recession (NO-recess), with unilateral IO recession (UNIL-recess), and with bilateral IO recession (BIL-recess). The success (version normalisation or at least 2 points upgrade in severity scale [0–4] in the operated eye), recurrence rates, and the evolution of the non-operated IO muscles were evaluated.

**Results:**

One hundred and ten patients were included – 53 NO-recess, 26 UNIL-recess, and 31 BIL-recess. Medial rectus muscle posterior fixation sutures surgery (PFS) was performed in 88.2% of patients for esotropia. A recession with graded anterior transposition was the weakening IO procedure. In the NO-recess group, 28 (52.8%) patients normalised their mild IOOA after PFS surgery alone. In the UNI-recess group, the success rate was 88.5%, with 16 (61.5%) patients showing worsened IO muscle of the fellow eye, which prompted additional surgery in 10 patients. In the BIL-recess group, all 31 patients improved the adduction pattern of the operated eye for an 80.6% success rate (6 improved marginally).

**Conclusion:**

Graded anterior transposition of the inferior oblique muscle effectively normalises versions. However, it’s frequent for a contralateral overaction to become manifest after unilateral IO surgery.

## Background

Primary inferior oblique muscle overaction (IOOA) is a common feature of primary horizontal strabismus, characterised by an overelevation of the adducted eye on version testing. It was reported to develop in approximately two-thirds of infantile esotropias and one-third of accommodative esotropias at an average age of 3.6 and 5.2 years, respectively [1]. A positive relationship was also found between the number of horizontal muscle surgeries needed and the incidence of IOOA in infantile esotropias [1]. Although primary IOOA is almost always bilateral, it can be unilateral or asymmetric, which makes the surgical decision difficult. When we decide on unilateral weakening surgery, we must be prepared for the appearance or worsening of the overaction of the contralateral inferior oblique muscle (IO) at a rate of up to 70%, depending on the surgical technique and the baseline versions [2–4]. Conversely, the presentation at the time of diagnosis is not static and can progress, with a unilateral IOOA becoming bilateral in 74.2% of patients before a surgical procedure [3].

Combining surgery for esotropia and an overacting IO muscle is often necessary. Previous studies have noted minimal effects of IO surgery on the primary position of gaze, suggesting that this factor doesn’t need to be included in planning the surgery for horizontal deviation [5, 6]. However, the influence of horizontal surgery on IO dysfunction is not fully understood. The purpose of this study was to answer the following questions: how do different patterns of symmetry and severity of IOOA evolve after esotropia surgery? What are the success and recurrence rates of simultaneous IO weakening?

## Methods

This study adhered to the tenets of the Declaration of Helsinki and was approved by our university institutional review board and ethics committee. The protocol and target patients of the study were, as previously published [7]. The medical records of consecutive patients who underwent surgery for esotropia between January 2009 and January 2020 at our hospital’s unit of Strabismus and Pediatric Ophthalmology (tertiary hospital) were reviewed retrospectively. Study inclusion criteria were as follows: patients with one of the following types of esotropia – infantile esotropia ([IE] present by six months of age), partially accommodative esotropia ([PAE] when accommodative factors contribute to but do not account for the total deviation in patients with a ≥ +2.00D of hyperopia and/or a > + 1.00D of anisohyperopia) and basic esotropia ([BE] a comitant esotropia that develops after age six months in patients with < + 2.00D of hyperopia); unilateral or bilateral overelevation in adduction (OEAd) caused by primary IOOA, presented at baseline or during the follow-up after esotropia surgery; at least one year of postoperative follow-up; clear and well-defined and documented medical records. Exclusion criteria were the presence of other types of esotropia; previous extraocular muscle surgery; other causes for OEAd, namely palsy of the superior oblique muscle or the contralateral superior rectus muscle, a dissociated vertical deviation, and orbital dysmorphisms with possible pulleys heterotopia.

All patients proposed for surgery comply with stereotypical orthoptic assessment pre- and postoperatively, carrying out photographic records that guarantee standardised and reliable data collection in the paired follow-up of the patient. All patients were evaluated for best-corrected visual acuity, version and duction examinations in full diagnostic positions, horizontal and vertical angle of deviation measurements using the alternative prism cover test and Krimsky method at both distance (6 m) and near (33 cm) fixation in primary position, synoptometer chart, and assessment of total cycloplegic refraction. The severity of IOOA was graded as + 1 to + 4 according to the upward and extorsion movement in adduction on ocular version testing [8]. An interocular difference of IOAA of > + 1 was considered an asymmetrical presentation. A review of each patient’s complete record was then performed. Before surgery, all patients were placed in total cycloplegic refraction and patching as needed by the type of deviation and for amblyopia treatment. Operative notes were reviewed for the type of procedure, intraoperative findings, technique, and complications. Examinations were scheduled 1–2 days before surgery and were repeated postoperatively on day 3 and 1, 6, and 12 months after surgery and annually after that.

The weakening procedure for the IO was carried through a recession with graded anterior transposition, performed simultaneously with the esotropia surgery as needed. A conjunctival traction 4/0 silk suture was placed at the inferotemporal limbus, and the globe was elevated and adducted. A fornix conjunctival incision was performed approximately 8 mm posterior to the limbus. After careful Tenon’s dissection, the IO muscle was hooked and isolated under direct visualisation. The muscle was secured with a full thickness 6 − 0 double-needle polyglactin suture, cut near its insertion, and sutured to the sclera adjacent to the temporal border of the inferior rectus (IR) muscle insertion (0–2 mm temporal and 0–3 mm posterior). The orientation line of the final insertion of the IO was oblique between the axis of the insertion and the lateral border of the IR, keeping the posterior fibres of the IO more posteriorly, with a distance of only 1 to 2 mm between the two ends. Finally, the conjunctiva was closed with an 8 − 0 polyglactin suture. Similarly to what was described by Guemes and Wright [9], the anterior transposition was classified by its relationship to the IR insertion: 0 mm - at the insertion, 1 mm posterior, 2 mm posterior, 3 mm posterior. The more the inferior oblique overacted, the more anterior the placement of the new insertion, corresponding to the scale + 1 to + 4 with the insertion 3 to 0 mm, respectively. Patients with ≥ +2 of IOOA were usually selected for surgery, although milder cases (+ 1) could be candidates for lower-dose surgery on a case-by-case basis, according to the surgeon’s experience. V patterns and vertical deviations in primary position also influenced the decision for surgery.

The enrolled patients were divided into three groups according to the baseline performed surgery: patients that underwent surgery for esotropia without IO recession (NO-recess), with simultaneous unilateral IO recession (UNI-recess), and with simultaneous bilateral IO recession (BIL-recess). The final success rate was defined as a normalisation of the versions or a grade improvement ≥2 in the IOOA severity scale on the operated side. The post-surgical evolution of the non-operated IO was also evaluated. Patients with a final trace of IOOA were also classified with a + 1 due to the subjectivity inherent to the scale.

Descriptive statistics are presented with absolute counts and proportions or with median and percentile 25–75 values, as appropriate. To compare differences across the three types of surgery performed (NO-recess, UNI-recess, and BIL-recess), we used chi-squared or Fisher’s exact tests for categorical variables and Kruskal-Wallis tests for continuous variables, given the asymmetry of the distributions. The significance level was set at α = 0.05. All data were analyzed using SPSS version 26 (SPSS Inc, Chicago, IL).

## Results

One hundred and ten patients met the inclusion criteria, presenting an esotropia with an IOOA – 27 (out of 67, 40.3%) cases of IE; 39 (out of 180, 21.7%) cases of PAE; and 44 (out of 157, 24.8%) cases of BE. The median age at the first surgery was 4,50 years (p25-p75, 3.0–6.0), with a prevalence of 61.8% for the feminine gender. Globally the preoperative horizontal deviation was ≥45 prism diopters (PD) in 81.6% of patients for near and ≥40 PD in 73.9% of patients for distance. The majority (n = 97, 88.2%) of patients underwent medial rectus (MR) muscle posterior fixation sutures to the sclera (PFS) without a recession as first-line surgery to correct esotropia [7] (versus n = 13, 11.8%, MR recession surgery). The median follow-up time was 7.0 years (p25-p75, 4.0–10.0).

The clinical features of the three study groups – 53 patients on NO-recess, 26 on UNI-recess, and 31 on BIL-recess – are summarised in Tables 1 and 2. In the NO-recess group, most patients had an asymmetrical and discrete IOOA (+ 1 unilateral), with a median vertical deviation of 4 PD in the primary position. Thirty-eight (71.7%) patients post-surgery remained bilaterally stable or improved. Twenty-eight (52.8%) patients normalised their unilateral + 1 IOOA after PFS surgery alone. In contrast, fourteen (26.4%) patients showed a unilateral worsening (just one patient worsened bilaterally), with IO surgery performed in 5 of these cases (all had + 1 IOOA at the beginning) in a second procedure. At the end of the follow-up, 5 cases of + 2 IOOA (all had + 1 IOOA at baseline) were proposed for additional surgery.

**Table 1:** Preoperative features and outcomes in the three groups of inferior oblique surgery

	NO-recess (n = 53)	UNI-recess (n = 26)	BIL-recess (n = 31)	p-value
Age at surgery, years (median, p25-p75)	5.0 (3.0-7.5)	4.5 (3.0–6.0)	4.0 (3.0–5.0)	0.181
Sex, female (n, %)	30 (56.6)	17 (65.4)	21 (67.7)	0.546
Follow-up, years (median, p25-p75)	7.0 (4.0–10.0)	5.5 (3.8-9.0)	7.0 (3.0–10.0)	0.795
Visual acuity, decimal (median, p25-p75)				
OD	0.8 (0.8-1.0)	0.8 (0.8-1.0)	0.8 (0.8-1.0)	0.997
OS	0.8 (0.6-1.0)	0.9 (0.8-1.0)	0.8 (0.8-1.0)	0.543
Type of esotropia (n, %)				
Infantile	12 (22.6)	8 (30.8)	7 (22.6)	0.840
Partially accommodative	18 (34.0)	8 (30.8)	13 (41.9)	
Basic	23 (43.4)	10 (38.5)	11 (35.5)	
Vertical deviation in pp, PD (median, p25-p75)				
Near	4.0 (0.0–9.0)	8.0 (4.0–10.0)	8.0 (6.0–10.0)	0.169
Distance	4.0 (0.0–8.0)	8.0 (4.0–10.0)	8.0 (4.3–10.0)	0.034
Inferior oblique pattern, baseline (n, %)				
Normal – 0 OU	8 (15.1)	0 (0.0)	0 (0.0)	< 0.001
Unilateral	31 (58.5)	18 (69.2)	0 (0.0)	
Bilateral	14 (26.4)	8 (30.8)	31 (100.0)	
Inferior oblique variation (n, %) *				
Stable or better – OU	38 (71.7)	10 (38.5)	31 (100.0)	< 0.001
Worse – OD and/or OS**	15 (28.3)	16 (61.5)	0 (0.0)	
Reintervention (n, %)	5 (9.4)	10 (38.5)	2 (6.5)	0.003
Inferior oblique pattern, final (n, %)				
Normal – 0 OU	29 (54.7)	16 (61.6)	20 (64.5)	0.368
+ 1 – OD and/or OS	19 (35.9)	5 (19.2)	9 (29.0)	
+ 2 – OD and/or OS	5 (9.4)	5 (19.2)	2 (6.5)	
Elevation deficit (n, %)	0 (0.0)	1 (3.8)	1 (3.2)	-
Additional surgery (n, %)	5 (9.4)	3 (11.5)	1 (3.2)	0.444

**Legend**: pp, primary position; p25-p75, percentile25-percentile75; OU, bilaterally; OD, right eye; OS, left eye; OD and/or OS, unilaterally or bilaterally

* After the first surgery

** Just one patient in the NO-recess group got worse bilaterally

**Table 2:** Baseline grade of inferior oblique overaction per eye in the three study groups

Grade of IOOA (n, %)	No-recess (n.53)		UNI-recess (n.26)		BIL-recess (n.31)	
	OD	OS	OD	OS	OD	OS
0	24 (45.3)	23 (43.4)	10 (38.5)	8 (30.8)	0 (0.0)	0 (0.0)
+ 1	23 (43.4)	22 (41.5)	5 (19.2)	1 (3.8)	1 (3.2)	1 (3.2)
+ 2	5 (9.4)	8 (15.1)	8 (30.8)	10 (38.5)	14 (45.2)	16 (51.6)
+ 3	1 (1.9)	0 (0.0)	3 (11.5)	7 (26.9)	16 (51.6)	14 (45.2)
+ 4	0 (0.0)	0 (0.0)	0 (0.0)	0 (0.0)	0 (0.0)	0 (0.0)

**Legend**: OD, right eye; OS, left eye

In the UNI-recess group, most patients had unilateral IOOA (69.2%). The remainder were bilaterally asymmetrical (e.g., + 2 or + 3 OD and + 1 OS), having been operated on the eye with the highest grade. Preoperatively, they presented a median vertical deviation of 8PD. Despite all patients having effective surgery on the operated eye, 16 (61.5%) showed worsening of the IO pattern on the contralateral eye. In ten of these cases (at baseline, 5 had + 1, 5 had 0), the worsening prompted further surgery, which was effective. At the end of the follow-up, three patients were waiting for additional surgery – 2 for unilateral + 2 IOOA (0 at baseline) and 1 for an under-elevation in abduction in the unilaterally operated eye (in this case, the new insertion was placed parallel, and slightly anterior to the IR insertion).

In the BIL-recess group, patients were mostly + 2 or + 3 symmetrical (71.0%) at baseline. When asymmetrical, the difference was + 1 degree. Preoperatively, they presented a median vertical deviation of 8PD. After the first surgery, all 31 patients (100%, 62 eyes) showed improved adduction. In two cases (1 bilaterally and one unilaterally) with only marginal improvement (+ 3 to + 2), the IOs were revised with further refinement. The bilateral case had a fibrotic capsule surrounding the IO with adhesions to the original insertion that were released. There was a case of discrete under-elevation in abduction, with almost normal versions, for which additional surgery was not proposed.

The surgical success rate (i.e., normalisation of the versions or a grade improvement ≥2 in the IOOA severity scale on the operated side) was 88.5% in the UNIL-recess group and 80.6% in the BIL-recess group. At the end of follow-up, 48 patients (90.6%) in the NO-recess group, 21 patients (80.8%) in the UNIL-recess group, and 29 patients (93.5%) in the BIL-recess group had normal bilateral versions or only mild overaction without surgical criteria. No operative complications were recorded.

## Discussion

There are a variety of procedures described that are effective in reducing inferior oblique muscle overaction [10–17]. The different published series vary from one another in short and medium-term success and especially in complication rates. Recurrent IOOA ranges from 15% after IO recession to 53% after IO disinsertion and 59–75% after IO muscle myectomy at the insertion [10, 14, 18]. Persistent IO overaction has been reported in 10–16% of the subjects after IO muscle anterior transposition [12, 18]. Something to consider when comparing series has to do with the study population, type of horizontal deviation, and particulars of the technique that can largely influence the final result.

Our surgical approach to this study cohort was effective, achieving complete normalisation of the versions bilaterally in 50 to 60% of cases or maintaining mild or near-normal overaction unilaterally in 80 to 94% of cases. All the operated oblique muscles obtained a stable improvement or normalisation of their overaction. No recurrences (as a return to the initial state) were recorded during a median follow-up period of 7 years. In a previous study, the recurrence of IOOA was detected on average three years after the initial surgery, emphasising the need for extended postoperative follow-up for its validity [1].

In 61.5% (16/26) of the patients who underwent unilateral surgery, an overaction of the non-operated fellow eye became manifest or worsened its previous mild dysfunction. On the other hand, 50% (13/26) of these patients will need to be reoperated. This report aligns with earlier studies in infantile and accommodative acquired esotropia patients [1, 4]. Kushner postulated that this might be partly due to the anti-elevating effect of the recessed IO causing an apparent overaction of the contralateral inferior oblique muscle by fixation duress [19]. The anterior transposition procedure changes the vector of forces by moving the new insertion toward the IR insertion [10]. Anteriorization of the posterior fibres of the IO muscle, mainly if more than 1 mm anterior to the IR insertion, has produced a high incidence rate of postoperative hypotropia and limited elevation in abduction, resulting in a Y or V pattern that mimics IOOA [19, 20]. Although plausible, this appears not to be the case in the current study, where the graded anterior transposition avoided the postoperative elevation limitation yet effectively corrected both mild and severe IOOA, as was previously described [9, 21]. The current technique has some different but important details, namely placing the new insertion obliquely relative to the insertion of the inferior rectus muscle, thus avoiding the J deformity, and with the two ends bunched together by 1-2 mm. It also is essential to place the sutures and cut the IO nearest to its tendon insertion, avoiding muscle resection that will create a future restriction to elevation.

Additionally, it has been proposed that the neurofibrovascular bundle connection to the orbital apex allows it to function as an ancillary IO origin, limiting the elevation of anteriorly transposed IO [19, 22]. Still, its colocation within the mechanically robust tissue of the IO pulley, rather than its origin, explains the sharp inflexion in the path of a transposed IO [23]. Performing the surgical exposure and dissection of the IO sheath, which disinserts the orbital layer from its pulley, will alter its mechanical action, even without further manipulations [23]. This is important to consider for the variability in the outcome of IO surgery among studies.

On the other hand, identical bilateral IO muscle recession [13] and myectomy [24] procedures on eyes with asymmetric degrees of IOOA were reported to produce the same percentage of symmetric results as the same surgery performed on patients with symmetric overactions, being self-adjusting and capable of achieving relatively symmetric versions. We obtained similar results in patients operated bilaterally, reaching normal bilateral versions in 93.5% of cases or only mild or trace overaction. Although, most of our cases (70.1%) presented symmetry in the preoperative overaction.

Our clinical opinion is that mild overacting IO can improve with medial rectus PFS surgery alone for the esotropia. Our results confirm that 52.8% (28/53) of the patients improved without IO muscle surgery. Wilson and Parks reported similar findings [1], in which the versions normalised in 12 patients with mild over-elevation in adduction after only horizontal muscle surgery. Unlike other weakening procedures, the PFS mode of action is likely due to the disruption of the MR pulley dynamics with its mechanical restriction and stretching against its anterior bony fixation, consequently decreasing ocular duction [7, 25–27]. Evidence exists for mechanical intercoupling among pulleys [28] and stereotypic shifts of rectus pulleys during gaze shifts [29]. In particular, the IR pulley shifts nasally in supraduction, suggesting mechanical coupling to the IO [30]. It may be a way to explain this relationship between PFS surgery and the improvement of slight over-elevations in adduction.

## Conclusions

Our study shares the limitations inherent in a retrospective study and lacks a control group. Despite standardisation in protocols, surgical decisions may not be completely homogeneous across the cohort. Also, the degree of torsion variation, the prevalence of the V-pattern, and its collapse with surgery were incomplete data in the records. As such, they were not presented, which may constitute a limitation. Regardless, this study allows drawing some conclusions. It is prudent to be patient in cases of mild IOOA, especially if undergoing PFS of the MR for esotropia. The graded anterior transposition of the IO muscles effectively reduces and corrects moderate to severe IOAA without limiting elevation. In apparently unilateral overaction of the inferior oblique muscle, a careful search should always be done in the fellow eye since it is frequent for a contralateral overaction to manifest after unilateral IO surgery. Bilateral surgery should be considered even in cases of asymmetrical overaction.

## Data Availability

Raw data for datasets are not publicly available to preserve individuals’ privacy under the European (and Portuguese) General Data Protection Regulation but are available from the corresponding author upon reasonable request.

## Abbreviations

BE: Basic esotropia
BIL-recess: Patients who underwent esotropia surgery with a bilateral inferior oblique recession
IE: Infantile esotropia
IO: Inferior oblique muscle
IOOA: Primary inferior oblique muscle overaction
IR: Inferior rectus
MR: Medial rectus
NO-recess: Patients who underwent esotropia surgery without an inferior oblique recession
OEAd: Overelevation in adduction
PAE: Partially accommodative esotropia
PD: Prism diopters
PFS: Posterior fixation sutures surgery to the sclera
UNIL-recess: Patients who underwent esotropia surgery with a unilateral inferior oblique recession
